# Increasing Probability of Heat-Related Mortality in a Mediterranean City Due to Urban Warming

**DOI:** 10.3390/ijerph15081571

**Published:** 2018-07-25

**Authors:** Andri Pyrgou, Mat Santamouris

**Affiliations:** 1 Energy, Environment and Water Research Center, The Cyprus Institute, P.O. Box 27456, Nicosia 1645, Cyprus; 2 The Anita Lawrence Chair in High Performance Architecture, School of Built Environment, University of New South Wales, Sydney 2052, Australia

**Keywords:** urban heat island, heatwaves, ozone, PM_10_, humidity, health, relative risk

## Abstract

Extreme temperatures impose thermal stress on human health, resulting in increased hospitalizations and mortality rate. We investigated the circulatory and respiratory causes of death for the years 2007 to 2014 inclusive for the urban and rural areas of Nicosia, Cyprus under urban heatwave and non-heatwave conditions. Heatwaves were defined as four or more consecutive days with mean urban daily temperature over the 90th percentile threshold temperature of the eight investigated years. Lag period of adverse health effects was found to be up to three days following the occurrence of high temperatures. The relative risk (RR) for mortality rate under heatwave and non-heatwave conditions was found taking in consideration the lag period. The results showed the increase of mortality risk particularly for men of ages 65–69 (RR = 2.38) and women of ages 65–74 (around RR = 2.54) in the urban area, showing that women were more vulnerable to heat extremities. High temperatures were also associated with high ozone concentrations, but they did not impose an excess risk factor, as they did not reach extreme values. This analysis highlights the importance of preparing for potential heat related health impacts even in Cyprus, which is an island with frequent heatwaves.

## 1. Introduction

Heatwaves (HWs) is a climatological phenomenon associated with high temperatures and increased thermal stress imposed on human health. Global mean temperatures are expected to rise by the end of this century, leading to an increase of the intensity, duration, and frequency of heatwaves. The impact of heatwaves on humans and natural systems include the increase of energy consumption, decreased air quality, intensification of droughts, and degradation of human health [1,2,3,4,5,6].

Urban heat island (UHI) is a microscale phenomenon that is associated with the rapid urbanization and industrialization, with the main characteristic being high urban temperatures that pose thermal stress on people. Several studies have explored the synergistic interactions between these two phenomena and revealed an intensification of the UHI phenomenon under heatwave conditions [7,8,9]. During heatwaves, mortality rate increases exponentially and the synergy with UHI constitutes it more harmful for urban residents. The reasoning is that the cool relief during the night is minimized due to the prolonged high urban temperatures caused by UHI phenomenon.

The escalation of temperature leads to heat waves whose consequences may become fatal for humans. The 2003 heat wave in western Europe resulted in over 70,000 heat-related deaths [10]. Chen et al. (2015) revealed that heat waves were correlated with higher mortality risks, resulting in a 24.6% increase in total mortality, 46.9% increase in cardiovascular mortality, 32% increase in respiratory mortality, 51.3% increase in stroke mortality, 63.4% increase in ischemic heart disease mortality, and 47.6% increase in chronic obstructive pulmonary disease mortality [11]. Other studies also reinforce the argument that women, elderly, and people with lower education level were more vulnerable during heat waves [11,12,13,14,15].

The development of early warning systems, improvement of health care services and change in infrastructure may assist in the mitigation of heat-related health effects [16]. Overcrowded and densely built urban areas affect urban ventilation and outdoor thermal comfort. Higher outdoor temperatures have a direct impact on indoor comfort conditions. The human body may efficiently regulate the heat to cope with thermal stress. However, when exceeding certain limits, thermal discomfort occurs and physical and mental activities are impaired with detriment to health. Higher relative humidity present at extreme heat waves may reduce the body’s ability to cool down by sweating [17]. Zhang et al. (2014) noted that absolute humidity—a direct measure of actual moisture in air—was the second most common predictor for total heat related mortality in four United States (U.S.) cities and proposed its consideration in future heat-health studies because it reflects the psychologically stressful heat exposure [18]. Additionally, hyperthermia risks increase with the extension of duration of a heat wave as a person’s tolerance of high temperatures decreases over time [17]. Exposure to high temperatures causes an increase in blood viscosity and blood cholesterol levels therefore exposure timeframe is a significant regulator when observing weather-related effect on human health. During heatwaves the cardiovascular system is affected; the blood pressure drops and the heart beat faster and more irregularly, heightening the risk for cardiac failure. Moreover, small blood clots can also form in blood vessels, cutting off normal blood flow to other parts of the body. When estimating the impact of weather on mortality, it is important to observe the exposure-response curve, lag structure, and temperature metric. Studies found a strong heat-related mortality association for same and previous-day exposure [19].

The eastern Mediterranean is part of the wider region the Middle East and North Africa with increasing population and multiple environmental and socioeconomic stresses that is projected to be severely affected by anthropogenic climate change, manifesting in increased dryness and the intensification of heat extremes [20,21]. Air pollution has gained a growing interest the past decades due to its negative effects on human health; specifically cardiovascular and respiratory related diseases [22,23,24,25,26,27]. Especially in metropolitan areas, environmental conditions may worsen, with a combination of increasing heatwaves and reduced air quality [28]. For example, the number of hot days in Nicosia, the capital of Cyprus (when maximum temperature is higher than 38 °C) may double [29], and ozone concentrations could constantly exceed the European Union’s (EU) air quality standard of 60 ppb or 120 μg/m^3^ for eight consecutive hours [30,31]. Cities that are already very hot need to implement substantially enough strategies to deal with the increased heat extremities [16]. Heaviside et al. (2016) examined the heat-related mortality in Nicosia from 2004 to 2009 inclusive and found that mortality rate increased steeply with temperature increase and specifically for an increase of 1 °C over baseline temperature the estimated heat-related mortality increased by 24%, and for a 5 °C temperature increase the heat related mortality increased by 133% [32]. Moreover, the same study statistically investigated the confounding effect of PM_10_ on mortality rates, concluding that it had a negligible effect on the exposure-response relationship for temperature-related mortality in Cyprus [32].

The purpose of the present study was to (i) present the seasonal mortality in Nicosia due to circulatory and respiratory causes in urban and rural areas, (ii) observe how temperature, absolute humidity, ozone, and PM_10_ concentration levels affect mortality rates in the summer with the cross correlation function, and (iii) compare the relative risk of mortality rates in the urban and rural areas of each age group based on the mean daily temperature and the exposure timeframe to extreme conditions. This study aims to improve earlier studies [32] investigating similar study periods by suggesting a different approach to determining the lag period of temperature and mortality rate. Moreover, it examines the effect of urban warming and related relative risk factors under urban HW conditions to identify how the urban built environment, and consequently, the UHI phenomenon impose excess heat stress on residents. Furthermore, this study adds to the literature as it examines the absolute humidity parameter, whereas previous study by Heaviside et al. (2016) only examined seasonality and relative humidity [32].

## 2. Methods

### 2.1. Study Area and Datasets

Cyprus is an island in the eastern basin of the Mediterranean Sea of area 9251 km^2^. One urban meteorological station (35.1653° N, 33.3550° E) in the residential area of Nicosia was chosen for investigation. Nicosia is a medium size city situated in the centre of Cyprus with population about 330 thousands, including the suburban areas [33]. Urban areas were defined according to the geo-codes of the Statistical service of Cyprus as the centre of Nicosia city and the following suburban areas; Agios Dometios (north-west of centre), Egkomi Lefkosias (west), Strovolos (south-west), Lakatameia (south), Latsia (south), and Geri (south-east). The north and north-east side of Nicosia is occupied and no data is available. Rural areas include all of the villages in the rest of the district of Nicosia.

Data were obtained for an eight-year time period, from 2007 to 2014 inclusive. Hourly meteorological (temperature (°C), relative humidity (%)) measurements were obtained from the Republic of Cyprus Ministry of Agriculture, Rural Development and Environment (MARDE). Daily mortality counts were obtained by the Health Monitoring Unit of the Ministry of Health of Cyprus. These data were explored based on exposure timeframe, vulnerability related to gender or age, cause of death, and confounding with heatwaves in terms of intensity and duration. Hourly air quality data (ozone (μg/m^3^) and PM_10_ (μg/m^3^)) were obtained from the Ministry of Labour, Welfare and Social Insurance.

### 2.2. Methodology

The applied methodology consisted of the following steps:

Observation of Mortality Rate

Mortality rates were obtained for circulatory and respiratory causes for years 2007–2014. Circulatory causes of death included ischemic heart diseases (I20–I25), cerebrovascular diseases (I60–I69), other heart diseases (I30–I51), and other circulatory diseases (I00–I15, I26–I28, I70–I99). Respiratory causes of death included influenza (J00–J99), pneumonia (J12–J18), chronic lower respiratory diseases (J40–J47), and other respiratory causes (J00–J06, J20–J39, J60–J99). Mortality rate was observed with respect to area (urban or rural), season, age group, and gender. Pyramid plots were utilized to show graphically the most sensitive age group and gender per season. Seasonal division was preferred to exclude deaths due to winter’s lower temperatures (influenza and pneumonia) and to emphasize on summer mortality rates that could be caused by extreme heat conditions. The mortality rate was observed with respect to the population in urban and rural areas.

The following table summarizes the population for Nicosia urban and rural areas, according to each age group. According to the Table, 237,703 people live in the urban area, specifically 113,833 males (47.89%) and 123,870 females (52.11%), and 87,249 people live in the rural area, specifically 43,474 males (49.83%) and 43,775 females (50.17%). This table (Table 1) was used to determine the percentage of people dying from respiratory or circulatory causes with respect to the gender and the population of the investigated area.

Definition of Prolonged Extreme Hot Events and Lag Period

Prolonged extreme hot events—heatwaves were defined as the time periods when the mean daily temperature (using hourly temperature values) exceeded 31.1 °C (90th percentile threshold temperature in urban station for years 2007–2014 (all seasons included)) for four or more consecutive days. This definition was chosen after a sensitivity analysis with varying heatwave threshold temperatures and duration of events. Mean daily temperature was preferred over maximum daily temperature because of the adverse health effects of prolonged heat exposure that may lead to mortality. According to this definition for the year span 2007–2014, the following extreme heat events (81 days in total) were recorded and are listed in Table 2. The lag period was also considered in the determination of the heatwave days for further correlation with mortality rate resulting to the 81 days listed in Table 2 plus the three days lag period for each heatwave event and resulting to a total of 113 days.

The lag period of temperature that had an effect on mortality rate was found using the cross correlation function (CCF analysis) in RStudio and it was determined to be three days. In an individual, the lag period should not be regarded as a well-defined interval, as it may vary according to the magnitude of the temperature and individual characteristics, such as acclimatization habits to heat, genetic background, and income. Therefore, for the estimation of the probability of mortality rate related to a particular occurrence the individual characteristics (other than age and sex) of a person are ignored. For the cross correlation function in RStudio, the x-variable (x_t+h_), y-variable (y), and the number of lags (h = ±25) were previously determined. If the highest peak occurs at a negative h then *x* is considered a predictor of *y*-variable. The number of lags reflects the number of days before and after a cross correlation is evaluated for variables *y* and *x*.

Non-heatwave days were considered the days from June until September (included) that were not listed in Table 2 and were not considered as lag days of events (total of 863 non-heatwave days for the eight investigated years).

Cross Correlation of Mortality Rate and Other Parameters

In the previous section, the mortality rate was correlated with mean daily or maximum daily temperature via the cross correlation function (CCF) in Rstudio software. Ozone levels, PM_10_ levels, and absolute humidity were also investigated to find a cross correlation with either temperature and/or mortality rate. The results would indicate the effect of air quality upon mortality rate. Heaviside et al. (2016) did not observe any correlation between PM_10_ and mortality rate in Cyprus but Pyrgou et al. (2018) noted increased ozone levels under heatwave conditions in Nicosia [28,32]. Moreover, absolute humidity correlation with mortality rate could reflect the psychologically stressful heat exposure and the importance of consideration of apparent temperature and Humidex factors in future studies. Absolute humidity was calculated according to the following equations [34,35]:

(1) $$Absolute\;Humidity\;\left(AH\right)\;=\;2.16679\frac{gk}{J}\cdot \left(\frac{P_{ws}}{T\left[K\right]}\right)\;$$

where *T* is the temperature in Kelvin and *P_ws_* is the vapour pressure, which was calculated according to the equation defined by Wagner and Pruß (2002) to sufficient accuracy between 0 °C and 373 °C [34]:

(2) $$\theta \;=\;1-\frac{Τ\left[Κ\right]}{647.096Κ}\;$$

(3) $$\ln\left(\frac{P_{ws}}{220640hPa}\right)\;=\;\frac{647.095}{T}\left(C_{1}\theta +C_{2}\theta^{1.5}+C_{3}\theta^{3}+C_{4}\theta^{3.5}+C_{5}\theta^{4}+C_{6}\theta^{7.5}\right)$$

where *C*_1_ = −7.85951783, *C*_2_ = 1.84408259, *C*_3_ = −11.7866497, *C*_4_ = 22.6807411, *C*_5_ = −15.9618719, and *C*_6_ = 1.80122502.

Relative Risk of Mortality with Respect to Summer Temperatures

Relative risk analysis descriptive statistic was preferred in this study because it is not an inferential statistic, as it does not determine statistical significance. Specifically, relative risk indicates the risk of the outcome of an exposed group relative to an unexposed group using percentage values. The two groups must have the same number of people/participants. To calculate the relative risk (RR) of mortality that is caused by circulatory and respiratory causes for all age groups and sexes we used the followed expression:

(4) $$\;RR\;=\;\frac{\frac{nr\;of\;people\;who\;died\;under\;HW\;conditions}{nr\;of\;people\;exposed\;to\;HW}}{\frac{nr\;of\;people\;who\;died\;under\;non-HW\;conditions}{nr\;of\;people\;under\;non-HW\;conditions}}\;$$

The above equation was also used for determining the relative risk per area, age group, and for each gender. Since the number of non-heatwave days was larger than heatwave days we had more recorded deaths under non-heatwave conditions. The two groups were re-evaluated to be comparable for the relative risk analysis using percentage values.

## 3. Results and Discussion

Total mortality caused by circulatory and respiratory causes was 6880 people for the investigated period of eight years in Nicosia, Cyprus. The initial analysis of the area of residency showed that about 71.9% (4949) of deceased lived in urban and suburban areas. Table 3 shows the percentage of number of deaths per cause (circulatory or respiratory) for the urban and rural sites, respectively. According to the table, the leading circulatory cause of death was ischemic heart disease at both areas and higher mortality rate due to respiratory causes were observed in the urban area compared to the rural area. Overall for the investigated time period (eight years) 2.08% and 2.21% of the urban and rural total population died from circulatory and respiratory causes, an average of 0.27% per year.

**Figure 1:**
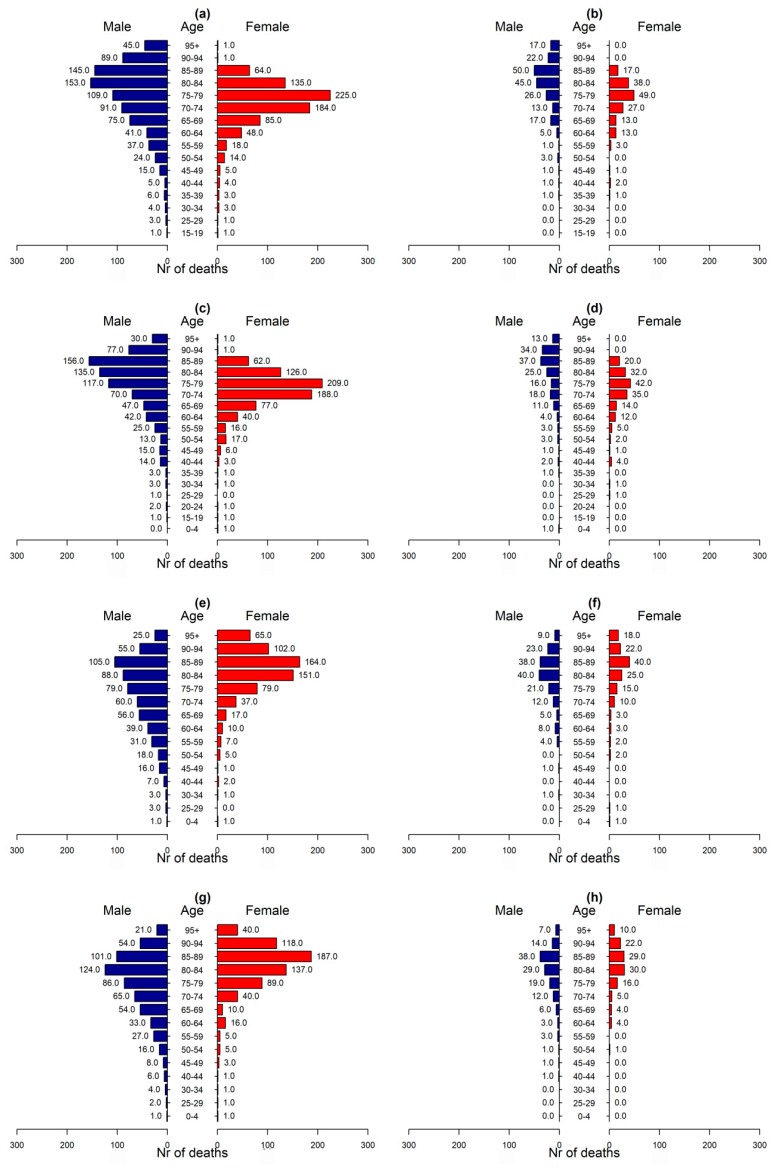
Mortality rate (nr of deaths—*x*-axis) by age group and gender (blue for male and red for female) for winter ((**a**) circulatory causes, (**b**) respiratory causes), spring ((**c**) circulatory, (**d**) respiratory), summer ((**e**) circulatory, (**f**) respiratory), and autumn ((**g**) circulatory, (**h**) respiratory) for years 2007–2014.

Figure 1 shows the total mortality by season, gender and age group for the two investigated causes of death. According to the data, recorded deaths from circulatory and respiratory causes were 1999, 1838, 1532, and 1511 for winter, spring, summer, and autumn, respectively. The highest mortality in all seasons was recorded for men of the age of 80–90 years old and women of the age of 75–85. According to Figure 1, there is an increased mortality of women of ages 75–85 as compared to other age groups. Also, according to the data, only half (49.9%) of deaths were women. The results also evidenced increased mortality due to respiratory diseases in winter and spring, when several various and influenza prevail in colder temperatures.

Focusing in the mortality rates during summer periods it was observed that older people, over 80, died suggesting an intolerance of human body to tolerate substantial heat. Physiological and behavioral adaptations may reduce mortality at higher temperatures. Adaptations to climate change and increased summer temperatures may also reduce risks. Nevertheless, even in Cyprus where heatwaves appear frequently and people are accustomed to heatwaves and have high levels of prevention awareness and air conditioning an increase in deaths can arise.

**Figure 2:**
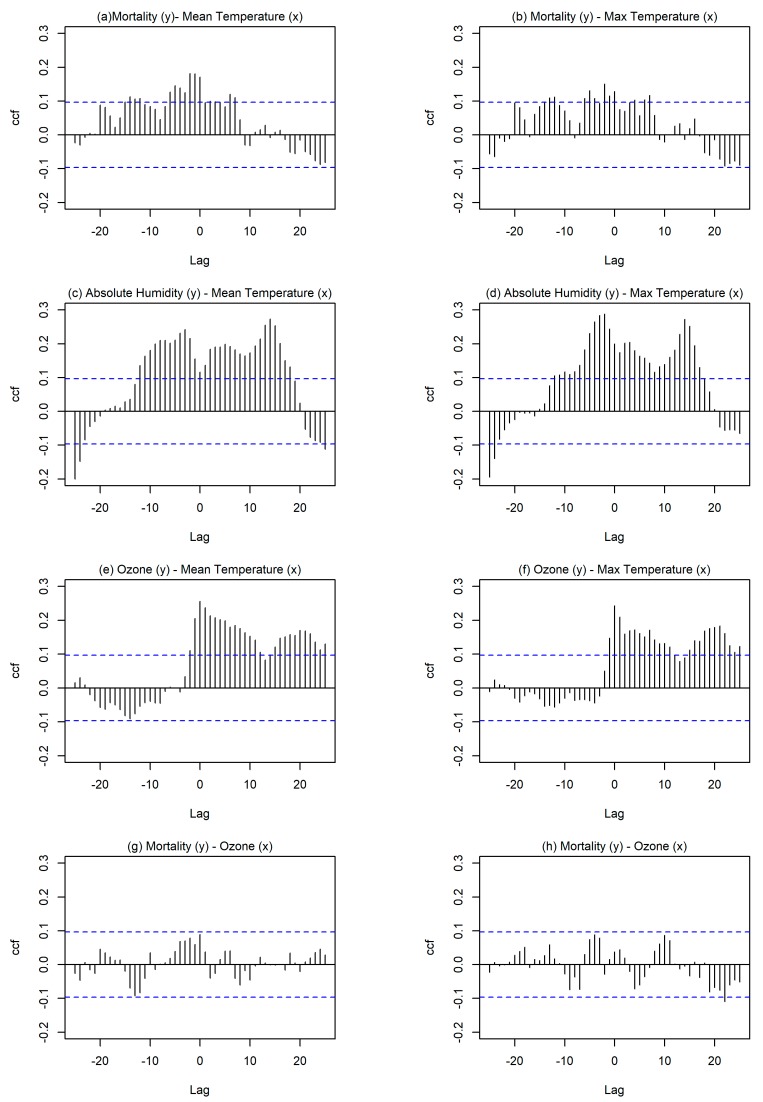
Cross correlation for (**a**) Mean daily temperature (*x*) and mortality (*y*); (**b**) Max daily temperature (*x*) and mortality (*y*); (**c**)Mean daily temperature (*x*) and absolute humidity (*y*); (**d**) Max daily temperature (*x*) and absolute humidity (*y*); (**e**) Mean daily temperature (*x*) and ozone (*y*); (**f**) Maximum daily temperature (*x*) and ozone (*y*); (**g**) Ozone levels (*x*) and mortality (*y*); and, (**h**) PM_10_ levels (*x*) and mortality (*y*). Dotted blue horizontal lines show 95% significance limits.

To understand the effect of the duration of specific parameters on the human body the following figure (Figure 2) was created for the investigated months June until September. Figure 2 examines the cross correlation of mortality rate with mean daily temperature (Figure 2a), maximum daily temperature (Figure 2b), mean daily ozone concentration (Figure 2g), and mean daily PM_10_ concentration (Figure 2h). It also examines the cross correlation of mean daily temperature with absolute humidity (Figure 2c) and with mean daily ozone concentration (Figure 2e) and the cross correlation of maximum daily temperature with absolute humidity (Figure 2d) and mean daily ozone levels (Figure 2f).

According to Figure 2, high mean daily temperatures had higher cross correlation with mortality rate suggesting that prolonged high temperatures negatively affect human health and impose thermal stress that leads to death. High cross correlation is observed when the bars exceed the horizontal blue lines. Moreover, in the case the highest lag period (h) is negative then it is considered that *x*-variable is a predictor of *y*-variable. Particularly, Figure 2a shows a lag period of exposure to the high mean daily temperature that could result to mortality. This emphasizes on the high probability risk of mortality even three days after exposure to high mean daily temperature conditions. A similar investigation was done for maximum daily temperature value with mortality rates (Figure 2b) showing a lower but still significant cross correlation, again with a time lag of three days. High cross correlation was observed for absolute humidity with mean and maximum temperatures and reached a maximum time lag of four days.

The time lag of ozone and temperature (mean daily and max daily) was 0 days, as expected because temperature acts as a catalyst for chemical processes involving ozone formation and these chemical processes are very fast. However, even though ozone concentrations were higher during heatwaves for Cyprus they did not have a significant effect on mortality rate, leading to the assumption that ozone concentration in Cyprus was high but it did not reach alarming levels to impose danger and mortality risk for the population. Moreover, PM_10_ values were evaluated with regard to mortality rate but no lag period was found regarding the high values of PM_10_ and mortality rate. The following table (Table 4) further adds to the preceding analysis as it contains the mean and maximum averaged daily values of the investigated parameters in the urban area for months from June to September for years from 2007 to 2014 inclusive—defined as non-heatwave period in the methods section (total of 863 days). According to Table 4, mean daily absolute humidity was higher under heatwave conditions, whereas relative humidity was lower. Moreover, the ozone values were higher under HW conditions but did not reach very high values for either investigated period (heatwaves or non-heatwaves), reinforcing the argument that they may have not imposed a risk factor for mortality rate in the urban area of Nicosia. Also, PM_10_ concentration levels reached the highest daily value (156.52 μg/m^3^) for a non-HW period therefore it could not be regarded as a determinant for increased mortality rate.

Following the proposed statistical analysis, for the investigation of the probability relative risk the mean daily temperature was chosen as the cause factor and lag period was set to three days, as determined from Figure 2a.

Heatwaves were previously defined in the methodology section and were listed in Table 2. The investigated time period that was used for the relative risk (RR) estimation presumed as heatwaves the time frame three days after the recorded events to include the effect of the lag period and resulting in a total of 113 days (81 days of heatwaves + 11 events × 3 lag days).

During the presumed heatwave periods (total of 113 days), 432 deceased were reported due to circulatory and respiratory causes, whereas for the non-heatwave periods (total of 863 days), 2595 deceased were reported due to circulatory and respiratory causes. The relative risk (RR) equation that was reported in the methodology section was used to determine the risk of death under heatwave (HW) conditions with respect to the risk of death under non-heatwave (non-HW) conditions.

(5) $$\;RR_{urban}\;=\;\frac{\frac{nr\;of\;people\;who\;died\;under\;HW\;conditions}{nr\;of\;people\;exposed\;to\;HW}}{\frac{nr\;of\;people\;who\;died\;under\;non-HW\;conditions}{nr\;of\;people\;under\;non-HW\;conditions}}\;=\;1.28\;$$

The relative mortality rate for all of the age groups and both genders under urban heatwave conditions and during the lag period of three days following heatwave conditions is RR = 1.28, showing that there is 1.28 times greater risk of mortality under heatwave conditions and the determined lag period. A relative risk of RR = 0.88 was found for all age groups and both genders under urban heatwave conditions in the rural areas. This shows the higher risk of mortality for urban population, which with the effect of urban heat island leads to higher urban temperatures which are more dangerous to human health.

A similar procedure was performed for each age group and each gender in the urban and rural areas mainly to indicate the importance of urban warming (higher RR in urban areas) and the vulnerability of gender and age group. The results are summarized in the following table:

According to the above table (Table 5), elderly and women have higher susceptibility to die under heatwave conditions. In urban area, men of age 65–69 under heatwave conditions had RR = 2.37 times the risk of death when compared to men of age 65–69 under non-heatwave conditions. The risk of death due to circulatory or respiratory causes among women of the same age (65–69) under heatwave conditions was RR = 2.57 times as high as the risk of death among women under non-heatwave conditions. Unexpectedly, the relative risk of death at higher ages, over 70 for men and over 75 for women decreased. Summarizing, according to the table above, in urban areas the most vulnerable age of death under heatwave conditions is 65–69 for men and 65–74 for women with a probability relative risk of RR = 2.38 and around RR = 2.54, respectively. There is a large variation between different age groups that may be based on other biological factors, but the important conclusion is that increased relative risk appears in almost all of the age groups under HW conditions.

A similar analysis was made for rural areas, but due to the small population of rural areas in Nicosia no clear conclusions were made. Nevertheless, it was noted an increased relative risk of mortality especially for the male population of ages 65–79 under urban heatwave conditions. The comparison of the relative risk columns for male and for female population in urban and rural areas revealed a higher risk in urban areas, which may be attributed to the higher temperatures that are caused also by the urban heat island effect observed in the city of Nicosia. The above table (Table 5) revealed two limitations of this study: the small population that was investigated for rural areas and the utilization of urban temperatures only, rather than also rural temperatures. The second limitation resulted due to the large area being considered as rural area and that the available rural stations would not reflect effectively the rural temperatures due to their large spread. Nevertheless, it appeared that urban warming was a main determinant of the increased mortality rate under heatwave conditions.

It should be noted that heatwave periods were determined according to the urban hourly temperatures. Heatwaves are defined as large scale phenomena that affect larger areas, whereas urban heat island is a microscale phenomenon. Previous studies showed that synergistic interactions of heatwaves with urban heat island phenomenon result in increased urban temperatures, urban warming, and overheating with heat island intensity being spatially heterogeneous in urban landscapes depending to the physical layout, urban design, land use mix, and street trees. Within city, different neighborhoods experience different rates of excess heat-related mortality and this variation may be correlated to poor housing conditions, poverty, impervious land cover, surface temperatures and residents’ hypertension [36,37].

It was fairly difficult to identify the local characteristics of each urban or rural area investigated as the spatial determinants and heat-related mortality is a non-linear relationship [36,37], and therefore the urban temperatures were preferred to examine mortality rate in the district of Nicosia. In addition, most people of rural areas work and/or travel to urban and suburban areas on a daily basis, therefore it is more appropriate to utilize urban temperatures. It was expected that excess risk would be imposed to residents in urban areas as a result to urban warming and the higher surface temperatures. Other factors other than area that affect the mortality rate are the exposure to high temperatures in other locations, such as working outdoors, walking, or taking public transportation. The findings affirm the importance of neighborhood characteristics and social determinant in targeting heat-health incidents and suggest that planning and design strategies for UHI mitigation should target resources to improve conditions in neighborhood vulnerable to urban warming, rather than greater areas characterized as urban or rural.

Synergistic interactions of urban heat island effect and heatwaves in Nicosia reveal an increase of temperature up to 2 °C. These higher urban temperatures due to the two phenomena can negatively affect human health. Moreover, the results of this study agree with a previous study by Heaviside et al. (2016), where heat-related mortality in Nicosia and in the whole Cyprus were investigated per degree of temperature over the baseline temperature for years 2004–2009 [32]. Specifically, for higher temperatures there was an increase in mortality that is caused by circulatory and respiratory causes.

In Figure 1, the mortality rate was recorded for all age groups in all seasons for circulatory and respiratory causes. Comparing the mortality rate of Figure 1 with the relative risk of Table 5 is shown according to the figure that the majority of male population died between ages 80–89 and of female population between ages 70–79 for the investigated causes. But, under heatwave conditions (Table 5), younger ages were mostly affected, ages 65–74 showing that extreme temperatures do not only affect already susceptible population but people of younger ages.

## 4. Conclusions

This study illustrated the mortality rate that was caused by respiratory and circulatory causes during the warm months (June–September) in Nicosia, Cyprus between 2007 and 2014 inclusive. The analysis showed that the majority of male population died between ages 80–89 and of female population between ages 70–79 for the investigated causes in the summer. However, the relative risk analysis showed that under heatwave conditions people of ages 65–74 were more vulnerable to heat-related mortality. Moreover, people in the urban areas had a greater risk of heat-related mortality probably due to the higher temperatures and the intensification of the temperatures due to the urban heat island phenomenon.

In this study, heatwaves were defined as the events when for four or more consecutive days the mean daily temperature exceeded the 90th percentile of temperature of years 2007 to 2014 inclusive. Mean daily temperature was preferred as the degradation of human health is mostly affected by the prolonged exposure to substantial heating. Moreover, a cross correlation analysis in RStudio software revealed a three-day lag period of adverse effects of high temperatures. This lag period was included in the determination of the relative risk of mortality under heatwave conditions in urban and rural areas. The time lag of ozone and temperature (mean daily and max daily) was 0 days, as expected because temperature acts as a catalyst for chemical processes and these chemical processes are very fast. However, ozone concentrations did not have a significant effect on mortality rate because they were not that high to reach alarming levels and impose excess mortality risk for the population.

Additionally, the relative risk analysis showed increased relative risk for people of ages 65–74 under heatwave conditions and particularly higher risk for women. Population in the urban areas appeared to be more vulnerable to heat with higher mortality at extreme temperature.

The results of this study add to the literature as there are limited studies based on mortality rates in Cyprus, and particularly based on heat-related causes. This study uses the definition of heatwaves to present the large scale phenomenon occurring over a larger area of the island, but also the urban temperatures were used to identify the effect of urban heat island on higher mortality rates in the city. A limitation of this study is the lack of meteorological stations at every area of residency, therefore leading to the choice of only urban temperatures for the HW definition.

This study also agrees with Heaviside et al. (2016) by concluding a negligible effect of PM_10_ on mortality rates, and also determining a lag period of around three days for temperature and increased mortality rates [32].

Adaptation measures will need to be developed, particularly within urban areas, in order to cope with the expected future intensification of heat waves due to global climate change. Even though Cyprus is an island with frequent occurrence of heatwave events older people still appear to be not acclimatized to extreme heat and result in higher heat-related mortality. It is fairly difficult to predict to what extent a population may adapt to temperature increase in the future but a good heat-health warning system may minimize these high mortality rates.

## Figures and Tables

**Table 1:** Male and female population in urban and rural areas of Nicosia according to population census of year 2011.

Age Group	Urban Area			Rural Area		
	Male	Female	Total	Male	Female	Total
0–4	6204	5960	12,164	2557	2506	5063
5–9	5610	5417	11,027	2487	2339	4826
10–14	6046	5835	11,881	2808	2565	5373
15–19	7225	6827	14,052	3325	3038	6363
20–24	9645	9281	18,926	3914	3454	7368
25–29	11,314	11,409	22,723	3931	3729	7660
30–34	10,335	11,369	21,704	3419	3639	7058
35–39	8256	10,481	18,737	2879	3328	6207
40–44	7638	9817	17,455	2780	3054	5834
45–49	7263	8721	15,984	2644	2815	5459
50–54	7521	8480	16,001	2779	2716	5495
55–59	6417	7044	13,461	2576	2425	5001
60–64	6261	6642	12,903	2248	2203	4451
65–69	4709	5094	9803	1677	1710	3387
70–74	3840	4365	8205	1366	1561	2927
75–79	2705	3156	5861	953	1206	2159
80+	2842	3968	6810	1131	1486	2617
Total	113,833	123,870	237,703	43,474	43,775	87,249

**Table 2:** Dates of heatwave events according to 90th percentile threshold temperature of years 2007–2014.

Events	Date of Heatwaves
1	23 June 2007–29 June 2007
2	23 July 2007–1 August2007
3	23 June 2008–27 June 2008
4	19 July 2008–22 July2008
5	31 July 2008–9 August 2008
6	19 July 2009–28 July 2009
7	31 July 2010–3 August 2010
8	13 July 2012–20 July 2012
9	19 June 2013–23 June 2013
10	22 July 2013–30 July 2013
11	2 August 2013–10 August 2013

**Table 3:** Percentage of circulatory and respiratory causes of death in urban and rural areas for the eight investigated years.

Area	Cause of Death	Deaths (nr)	Percentage Per Urban/Rural Population (%)
Urban	Ischaemic heart disease	1309	0.55
	Cerebrovascular disease	882	0.37
	Other heart diseases	1147	0.48
	Other circulatory diseases	657	0.28
	Influenza	9	0.00
	Pneumonia	249	0.10
	Chronic lower respiratory diseases	227	0.10
	Other respiratory diseases	469	0.20
Rural	Ischaemic heart disease	482	0.55
	Cerebrovascular disease	336	0.39
	Other heart diseases	514	0.59
	Other circulatory diseases	290	0.33
	Influenza	1	0.00
	Pneumonia	58	0.07
	Chronic lower respiratory diseases	119	0.14
	Other respiratory diseases	130	0.15

**Table 4:** Mean and maximum daily averaged values of several parameters under heatwave (HW) and non-HW conditions for years 2007–2014.

Parameter	Maximum Value under Non-HW Conditions	Mean Value under Non-HW Conditions	Maximum Value under HW Conditions	Mean Value under HW Conditions
Temperature (°C)	33.74	28.15	35.08	31.67
Relative Humidity (%)	87.33	51.64	75.33	42.42
Absolute Humidity (μg/m^3^)	2.86	1.51	2.94	1.77
Ozone levels (μg/m^3^)	106.64	69.63	99.60	78.08
PM_10_ levels (μg/m^3^)	156.52	40.26	80.70	40.96

**Table 5:** Relative risk (RR) of mortality due to circulatory and respiratory causes for men and women of different age groups in urban and rural areas for years 2007–2014.

Age Group	RR in Urban Areas		RR in Rural Areas	
	Men	Women	Men	Women
0–59	1.19	1.44	1.54	0.86
60–64	1.17	-	0.91	1.93
65–69	2.38	2.57	1.34	-
70–74	0.89	2.49	1.83	0.64
75–79	1.33	0.75	1.63	-
80+	0.96	1.48	0.55	1.19

Note: - no deaths.
